# Immediate implant placement in infected extraction sockets: A scoping review of case selection, decontamination protocols, clinical outcomes and complications

**DOI:** 10.4317/medoral.28256

**Published:** 2026-06-28

**Authors:** Fernando Mauricio Espada-Salgado, María Mihaela Iuga, Bogdan Oprea

**Affiliations:** 1 Faculty of Health Sciences, Private University of Tacna, Tacna, Peru; 2 Department of Dental Morphology, Lucian Blaga University of Sibiu, Sibiu, Romania

**Keywords:** Dental implants, immediate implant placement, infected extraction socket, periapical pathology, scoping review.

## Abstract

**Background:**

This scoping review aimed to map the extent and nature of the evidence on immediate implant placement in infected extraction sockets, with emphasis on case selection, decontamination protocols, adjunctive interventions, clinical outcomes, and complications.

**Materials and Methods:**

This review was conducted in accordance with Joanna Briggs Institute guidance for scoping reviews and reported following PRISMA-ScR. Searches were performed in Scopus, Web of Science Core Collection, PubMed/MEDLINE, LILACS via BVS, SciELO, and ClinicalTrials.gov up to 17 March 2026. After removal of duplicates, 72 records were screened independently by two reviewers, and 24 clinical studies published between 2015 and 2025 met the inclusion criteria.

**Results:**

The included evidence comprised case reports, case series, retrospective studies, prospective cohort studies, one split-mouth study, and one randomized trial. Most studies evaluated infected sockets associated with endodontic or periapical lesions, whereas periodontal and mixed lesions were less frequently addressed. Across studies, atraumatic extraction, meticulous socket debridement, and achievement of primary stability were consistently reported as key procedural steps. Adjunctive measures included systemic antibiotics, chlorhexidine decontamination, bone grafts, barrier membranes, platelet concentrates, laser therapy, vestibular socket techniques, and immediate provisionalization. Although most studies reported favorable implant survival, marginal bone stability, and esthetic outcomes, the evidence was heterogeneous, largely observational, and frequently non-comparative.

**Conclusions:**

The available evidence suggests that immediate implant placement in infected extraction sockets may be feasible in carefully selected cases when meticulous debridement and adequate primary stability are achieved. However, due to the heterogeneity, limited comparative evidence, and variability in infection definitions and treatment protocols, no standardized clinical protocol can currently be recommended. Further well-designed comparative studies with standardized reporting are needed.

## Introduction

Immediate implant placement (IIP) has become an increasingly attractive treatment option because it shortens treatment time, reduces the number of surgical interventions, and may help preserve peri-implant tissue architecture. However, clinical outcomes in compromised sockets depend on local defect morphology, the possibility of achieving primary stability, and the clinician’s ability to control local contamination. These factors make treatment planning more demanding than in intact post-extraction sites [1].

Infected extraction sockets remain one of the most debated scenarios for IIP. Residual bacteria, granulation tissue, and apical or periodontal inflammatory lesions have traditionally raised concerns about impaired osseointegration and early implant failure. This concern is supported by a meta-analysis reporting a higher risk of failure for immediate implants placed in infected sites compared with non-infected sites [2]. Conversely, more recent evidence syntheses focused on periapical pathology have reported high survival rates and did not identify a clearly increased risk when meticulous debridement, careful case selection, and adequate implant stability were achieved [3,4].

Primary clinical studies also reflect this controversy. In a split-mouth study, Hita-Iglesias *et al*. reported lower survival for implants placed in sockets associated with chronic periapical pathology than for implants placed in healthy sockets [5]. In contrast, Zuffetti *et al*. found favorable outcomes for immediate implants placed in endodontically or periodontally infected sites in a multicenter retrospective cohort [6]. Prospective evidence has also suggested that immediate provisionalization may be feasible in acutely infected sockets after thorough socket debridement [7]. Similarly, a consecutive cohort study using a standardized decontamination protocol supported the clinical viability of this approach [8]. More recent observational studies have continued to report encouraging survival and peri-implant tissue outcomes in sockets with chronic periapical pathology and in compromised infected sockets treated with advanced reconstructive approaches, such as vestibular socket therapy [9,10].

Despite this growing body of evidence, the literature remains fragmented. Studies differ in their definitions of infection, eligibility criteria, debridement and decontamination procedures, use of antibiotics or regenerative materials, and outcome measures. Clinical, radiographic, esthetic, and patient-reported outcomes are also assessed inconsistently across studies. This heterogeneity limits direct comparison and makes it difficult to identify consistent indications, contraindications, and protocol elements for clinical decision-making. Therefore, this scoping review aimed to map the extent and nature of the available evidence on IIP in infected extraction sockets, with particular attention to case selection, decontamination strategies, complementary interventions, treatment outcomes, and complications.

## Materials and Methods

*Methodological approach*

This scoping review was conducted in accordance with the updated Joanna Briggs Institute (JBI) methodological guidance for scoping reviews [11]. Reporting followed the Preferred Reporting Items for Systematic Reviews and Meta-Analyses extension for Scoping Reviews (PRISMA-ScR) [12]. Consistent with JBI guidance for evidence mapping, no formal risk-of-bias appraisal was performed, and the findings were synthesized descriptively [11].

*Protocol and registration*

The review protocol was registered in the Open Science Framework (OSF) under the open-ended registration code pb3nh. The OSF record includes the protocol package and project documentation, supporting transparency and reproducibility throughout the review process.

*Review question and PCC framework*

The review question was developed using the Population-Concept-Context (PCC) framework. The population comprised human patients undergoing tooth extraction followed by immediate dental implant placement in infected extraction sockets. Eligible infectious conditions included pre-existing endodontic, periapical, periodontal, and endo-periodontal infections, as defined clinically and/or radiographically in the original studies.

The concept of interest was the evidence available on IIP in infected extraction sockets. Particular emphasis was placed on case selection criteria, debridement and decontamination protocols, adjunctive therapeutic measures, treatment outcomes, and complications. The context included oral surgery and implantology settings, such as private practice, hospital-based care, university clinics, and specialist implant centers.

Accordingly, the review question was: What is the extent and nature of the available evidence on immediate implant placement in infected extraction sockets regarding selection criteria, decontamination protocols, clinical outcomes, and complications?

*Eligibility criteria*

Original human clinical studies were considered eligible when they addressed immediate implant placement in infected extraction sockets and reported at least one relevant element related to case selection, diagnostic features of the infected site, debridement or decontamination, systemic or local antimicrobials, grafting or membrane use, implant survival or success, peri-implant tissue outcomes, marginal bone changes, esthetic outcomes, patient-reported outcomes, or biological or technical complications.

Eligible study designs included randomized clinical trials, non-randomized interventional studies, prospective and retrospective cohort studies, case-control studies, case series, and case reports. The review focused on studies published between 2015 and 2026. Records were excluded when they did not provide original clinical data, did not focus centrally on IIP in infected extraction sockets, corresponded to non-final manuscript drafts, were outside the predefined publication window, or could not be retrieved in full text.

The 2015-2026 publication window was selected to map contemporary clinical evidence reflecting current IIP protocols, modern implant surfaces, CBCT-based assessment, and the increasing use of adjunctive decontamination or regenerative strategies. These strategies included platelet concentrates, laser-assisted decontamination, vestibular socket therapy, ozone-based approaches, and guided bone regeneration. Earlier studies were considered relevant for historical background but were not included in the evidence map, as the objective was to characterize recent clinical practice and reporting patterns rather than the historical development of IIP.

*Information sources and search strategy*

Systematic searches were conducted in Scopus, Web of Science Core Collection, PubMed/MEDLINE, LILACS via BVS, SciELO, and ClinicalTrials.gov. The final search was run on 17 March 2026. Source-specific search strategies were adapted to the syntax and indexing characteristics of each database or register.

Search strings combined terms related to IIP with terms describing infected extraction sockets and related endodontic, periapical, and periodontal pathology. Representative terms included “immediate implant placement”, “immediate post-extraction implant placement”, “infected extraction socket”, “periapical lesion”, “apical periodontitis”, “endodontic lesion”, and “endo-periodontal lesion”. Multilingual variants were incorporated in regional sources when appropriate. Operational filters were applied in some interfaces, and the complete search strategies for all sources were documented in the OSF materials.

*Selection of sources of evidence*

All retrieved records were exported to Zotero for de-duplication. After duplicate removal, screening was conducted in Rayyan [13]. Two reviewers independently screened titles and abstracts, followed by full-text assessment of potentially eligible reports. Disagreements were resolved through discussion and, when necessary, through consultation with a third reviewer acting as adjudicator.

A total of 93 records were identified across databases and registers. After removal of 21 duplicates, 72 unique records were screened by title and abstract. Thirty-five reports were sought for retrieval, of which eight could not be obtained. Unavailable reports were documented after attempts to locate the full text through database links, publisher websites, institutional access, and manual searching.

Consequently, 27 full-text reports were assessed for eligibility, and 24 studies were included in the final review. Reasons for full-text exclusion were explicitly recorded, and the full-text eligibility decisions are summarized in Supplementary Appendix S1 (https://www.medicinaoral.com/medoralfree01/28256/Suppl1.pdf).

*Data charting*

Data were charted using a structured extraction form developed for this review. The variables included bibliographic characteristics, study design, clinical setting, sample size and clinical context, type of infection or pathology, selection criteria, local site features, debridement and decontamination procedures, complementary measures, implant loading protocol, follow-up duration, and reported outcomes.

Complementary measures included antibiotics, grafting materials, membranes, platelet concentrates, laser therapy, and ozone-based approaches. Reported outcomes comprised clinical, radiographic, esthetic, survival/success, patient-related, and complication outcomes.

*Synthesis of results*

Findings were synthesized descriptively in tables and narrative form. The review aimed to map the extent, characteristics, and heterogeneity of the available evidence rather than to estimate pooled effects. Therefore, no meta-analysis was undertaken, and outcomes were summarized according to the objectives of a scoping review and JBI recommendations [11].

To contextualize the robustness of the mapped evidence, the narrative synthesis considered study design and comparative structure. Descriptive reports and case series, retrospective observational studies, prospective observational studies, controlled comparative designs, and randomized clinical evidence were differentiated. This classification was used to describe the structure of the evidence base without performing a formal risk-of-bias appraisal.

## Results

*Study selection*

The searches across databases and registers identified 93 records. After removing 21 duplicates, 72 unique records were screened by title and abstract. Thirty-five reports were sought for full-text retrieval; however, 8 could not be obtained despite retrieval attempts. Therefore, 27 full-text reports were assessed for eligibility.

Of these, 24 studies met the inclusion criteria and were included in the review. Three reports were excluded for prespecified reasons: One was published outside the predefined time window, one corresponded to a manuscript draft or non-final publication, and one did not focus centrally on immediate implant placement in infected extraction sockets. The study selection process is shown in Figure 1. Details of the full-text eligibility decisions are provided in Supplementary Appendix S1 (https://www.medicinaoral.com/medoralfree01/28256/Suppl1.pdf).

*Characteristics of the included evidence*

The included studies were published between 2015 and 2025. Publications were more frequent from 2018 onward, with visible peaks in 2018 and 2023 (Figure 2). The evidence base was methodologically heterogeneous and included 8 descriptive reports (7 case reports and 1 case series), 7 retrospective observational studies, 7 prospective observational studies, 1 split-mouth controlled study and 1 randomized clinical trial. The studies were conducted in Europe, Asia, and Latin America, including Spain, Italy, Turkey, Egypt, Iran, India, China, Brazil, Chile and Paraguay.

Most studies focused on endodontic or periapical infections, particularly chronic apical periodontitis, asymptomatic apical periodontitis, acute periapical pathology, or radiographically defined periapical lesions. Fewer reports addressed periodontal infection, mixed endo-periodontal disease, or broader clinically infected sockets.

The anatomical context also varied across studies. Many reports involved anterior maxillary or esthetic-zone cases, whereas others included mixed maxillary and mandibular sites, full-arch mandibular rehabilitation, or mandibular molars. Comparative studies generally evaluated infected sockets against non-infected controls, while descriptive reports mainly illustrated complex regenerative or esthetic scenarios. The general characteristics of the included studies are summarized in Table 1.

*Clinical management strategies*

Atraumatic extraction, followed by careful socket curettage and debridement, was the most consistently reported procedural step across the included studies. In contrast, decontamination protocols and adjunctive management strategies varied considerably (Table 2, Figure 3). Chemical antisepsis was described selectively, most often through chlorhexidine-based disinfection protocols. Systemic antibiotics were also frequently used in protocol-driven studies and case series; however, the specific regimens, timing, and duration were not consistently reported.

Adjunctive regenerative procedures were commonly described. Guided bone regeneration, bone substitutes, collagen membranes, and other grafting approaches were mainly used in sockets with jumping gaps, buccal plate defects, sinus-related limitations, or high esthetic demands. Platelet-derived biomaterials, including platelet-rich fibrin (PRF), concentrated growth factor (CGF), and plasma rich in growth factors (PRGF), were also reported either as stand-alone adjuncts or in combination with grafting materials. More specialized approaches included Er,Cr:YSGG laser-assisted decontamination, ozonated olive oil, vestibular socket therapy using a 6-day protocol, and endoscopically assisted transcrestal sinus floor elevation.

Immediate provisionalization or immediate loading was reported in a subset of studies, particularly in anterior esthetic cases and in protocols designed to shorten treatment time. Nevertheless, restorative timing was not uniform across the evidence base. Reported strategies ranged from immediate provisional crowns to delayed prosthetic loading after several weeks or months. This variability in loading protocols further contributed to the heterogeneity of the mapped evidence.

*Clinical, radiographic, and esthetic outcomes*

Overall, the mapped evidence indicates that immediate implant placement in infected extraction sockets may achieve favorable clinical, radiographic, and esthetic outcomes when strict case selection and rigorous local management are applied. Most comparative and observational studies reported high implant survival in infected sockets. However, the interpretation of these findings should be cautious, as several studies were observational and protocols varied substantially. In studies with non-infected controls, survival, marginal bone behavior, and soft-tissue outcomes were often comparable between groups (Table 3).

Among the comparative studies, Zuffetti *et al*. [6] found similar cumulative survival rates for implants placed in infected and non-infected sites over medium-term follow-up. Muñoz-Cámara *et al*. [7] also found no significant clinical or radiographic differences after one year between implants placed in sockets with acute periapical infection and those placed in non-infected controls, with all implants restored using immediate provisional restorations. Similarly, Yang *et al*. [31] observed no significant five-year differences between mandibular molars with and without chronic apical periodontitis in terms of peri-implant bone density, bone mass changes, complications, or survival. Crespi *et al*. [19] described complete radiographic resolution of the apical area and 100% implant survival, regardless of whether reactive tissue was removed.

However, the findings were not fully consistent across analytical studies. In a split-mouth study, Hita-Iglesias *et al*. [5] reported significantly lower survival for implants placed in sockets associated with chronic periapical pathology than for those placed in healthy sockets. Çolak and Demirsoy [9] also reported generally high success rates in infected sockets, although outcomes were less favorable when immediate implant placement was combined with simultaneous sinus lift procedures. In the large retrospective study by da Silva *et al*. [24], chronic apical periodontitis was not significantly associated with implant failure. Instead, short implants and heavy smoking emerged as more relevant predictors of adverse outcomes.

Studies evaluating complementary strategies generally showed encouraging results. Crippa *et al*. [18] found no relevant increase in implant failure or marginal bone loss in infected sockets treated with Er,Cr:YSGG laser decontamination. Kakar *et al*. [8] observed survival above 95% using a protocol that combined laser decontamination, systemic antibiotics, grafting, and non-submerged healing. Elaskary *et al*. [10] reported 100% survival and stable hard- and soft-tissue outcomes in infected type II sockets managed with vestibular socket therapy. In the randomized clinical trial by Zohary *et al*. [25], platelet-rich fibrin (PRF) and, particularly, concentrated growth factor (CGF) were associated with better soft-tissue and patient-centered parameters than control treatment, while maintaining successful osseointegration.

Descriptive reports and case series also documented favorable clinical resolution in complex infected sockets. These included esthetic-zone cases, sockets with buccal plate defects, sinus-related anatomical limitations, and cases requiring immediate provisionalization. Follow-up in these reports ranged from 10 months to 5 years. Overall, these studies described successful osseointegration, stable peri-implant tissues, acceptable esthetic outcomes, and no major biological complications.

*Complications and evidence gaps identified during mapping*

Complications were inconsistently reported across the evidence base. When implant failures were described, they tended to occur early, mainly within the first year. Biological complications were uncommon in the included studies. Notably, retrograde peri-implantitis was not observed in the prospective cohort that evaluated immediate provisionalization in sockets with acute periapical pathology. Prosthetic complications were rarely detailed, although veneer fractures were reported in the long-term immediate-loading cohort by Anitua *et al*. [26].

The mapped literature showed marked heterogeneity in infection definitions, diagnostic thresholds, socket morphology, implant sites, complementary therapies, loading protocols, and outcome reporting. Together with the predominance of descriptive studies and non-randomized designs, this heterogeneity indicates that the evidence remains clinically promising but methodologically fragmented.

## Discussion

This scoping review mapped the available evidence on immediate implant placement in infected extraction sockets. Overall, the findings suggest that this approach may be feasible in carefully selected cases when strict local management is applied. However, the evidence remains heterogeneous and should be interpreted with caution. Most included studies were observational or descriptive, and protocols differed in diagnostic criteria for infection, socket morphology, decontamination procedures, complementary therapies, loading strategies, and outcome reporting. Earlier meta-analytic evidence suggested a possible increase in implant failure in infected sites, whereas more recent reviews indicate that this association is inconsistent and may depend on case selection, local anatomy, decontamination, and surgical execution [1-4].

A key interpretation is that infection should be regarded as a modifier of case complexity rather than as an absolute contraindication. From a clinical perspective, the main issue is not only whether infection is present, but whether the socket can be converted into a biologically controlled and mechanically stable implant bed. This may explain the differences observed across the included studies. Hita-Iglesias *et al*. reported lower survival in sockets with chronic periapical pathology [5]. In contrast, Zuffetti *et al*., Muñoz-Cámara *et al*., Kakar *et al*., Çolak and Demirsoy, and Elaskary *et al*. reported favorable outcomes when atraumatic extraction, thorough debridement, local decontamination, and adequate initial implant stability were achieved [6-10]. These findings suggest that pre-existing infection remains clinically relevant, although its effect is influenced by anatomical, patient-related, and procedural factors.

The term “infected extraction socket” encompasses different clinical and biological conditions. The mapped literature included asymptomatic chronic apical lesions, acute periapical pathology, endodontic infection, periodontal involvement, and mixed endo-periodontal lesions. These conditions were not always defined using standardized diagnostic criteria. Anatomical context also varied, ranging from anterior esthetic sites to posterior regions and mandibular molars. This distinction is clinically important because residual bone, implant stability, the need for augmentation, and the risk of esthetic or biological complications may differ substantially across sites. Therefore, the current evidence cannot be translated into a single protocol for all infected sockets.

Complementary measures were frequently reported, but no specific strategy can currently be considered superior. Across studies, socket debridement was combined with systemic antibiotics, antiseptic irrigation, grafting materials, collagen membranes, platelet concentrates, laser-assisted decontamination, ozone-based approaches, vestibular socket therapy, and immediate provisionalization. Case-based evidence showed favorable clinical and esthetic outcomes in selected infected sockets treated with atraumatic extraction, guided bone regeneration, leukocyte- and platelet-rich fibrin, fibrin-based biomaterials, and immediate or delayed prosthetic rehabilitation [14-17]. Laser-assisted decontamination and other additional procedures were also reported in cohort studies, prospective studies, and case reports [18-23]. These approaches may support local management, but they should not be interpreted as independently validated or standardized interventions.

The overall level of evidence remains limited. Most included studies were case reports, case series, or retrospective cohorts, whereas controlled prospective studies and randomized trials were less frequent. The retrospective study by da Silva *et al*. found that chronic apical periodontitis itself was not significantly associated with implant failure. Instead, short implants and heavy smoking were more relevant predictors of adverse outcomes [24]. Zohary *et al*. reported successful osseointegration in previously infected sites treated with platelet-rich fibrin or concentrated growth factor, with better soft-tissue and patient-centered outcomes in the concentrated growth factor group [25]. Other studies contributed evidence on immediate loading, prepared infected sockets, sinus-related periapical infection, platelet-rich fibrin-assisted esthetic-zone management, and mandibular molars with chronic apical periodontitis [26-31]. Taken together, these findings suggest that treatment outcomes may depend more on case characteristics and protocol execution than on infection alone.

Recent studies provide additional context. Yu *et al*. showed that immediate implant placement in mandibular molars with chronic periapical periodontitis was feasible and achieved complete short-term survival, although buccal horizontal bone loss was greater than after delayed placement [32]. Amato *et al*. reported comparable survival for immediately placed single implants with immediate provisional restorations in non-infected, acutely infected, and chronically infected esthetic-zone sites over medium- to long-term follow-up [33]. Donker *et al*. also reported favorable clinical, radiographic, esthetic, and patient-reported outcomes using a digital workflow in the maxillary esthetic zone [34]. These findings support feasibility in selected scenarios, but they do not justify routine or indiscriminate use.

Site-specific interpretation is essential. Evidence from anterior esthetic sites should not be directly extrapolated to posterior or molar sockets. Posterior sites often present wider sockets, limited septal support, and more complex defect morphology. Surgical approach may also influence peri-implant tissue preservation. Pitman *et al*. reported greater buccal bone preservation with flapless surgery than with flap elevation [35], while Wu *et al*. showed that surgical approach may affect buccal bone thickness and midfacial soft-tissue recession after anterior immediate implant placement [36]. Although these studies were not restricted to infected sockets, they remain relevant because infected sites often present thin buccal plates, deficient socket walls, or increased esthetic risk.

Implant survival alone is an insufficient marker of success. Most included studies emphasized survival or osseointegration, whereas fewer reported biological complications, soft-tissue stability, esthetic outcomes, prosthetic events, or patient-reported outcomes in a standardized manner. Recent systematic evidence suggests that immediate implant placement should be assessed not only by survival, but also by esthetic integration and soft-tissue stability [37]. Similarly, long-term evidence on immediately loaded implants placed in fresh sockets indicates that favorable survival may coexist with biological and technical complications over time [38]. Future studies should therefore include standardized reporting of marginal bone changes, peri-implant soft-tissue stability, esthetic outcomes, patient-reported outcomes, and biological and prosthetic complications.

This review has limitations. First, the included evidence was clinically and methodologically heterogeneous. Differences were observed in infection definitions, socket morphology, implant sites, decontamination protocols, complementary interventions, loading strategies, and follow-up duration. Second, the predominance of descriptive and observational studies limits the strength of clinical inference. Third, because formal risk-of-bias assessment is not mandatory in scoping reviews, the findings should be interpreted as evidence mapping rather than as certainty regarding effectiveness. Fourth, the restricted publication window may have excluded earlier studies that could have provided historical context. Finally, inconsistent reporting of esthetic outcomes, soft-tissue behavior, prosthetic complications, and patient-reported outcomes limited direct comparison across studies.

From a clinical perspective, the findings support selective rather than routine immediate implant placement in infected extraction sockets. Predictability appears greater when atraumatic extraction, thorough debridement, local decontamination, sufficient native bone, and adequate initial implant stability are achieved. Regenerative or decontamination procedures may be useful when indicated, but they should be selected according to defect morphology and biological requirements. Predictability may decrease in cases with severe socket destruction, simultaneous sinus-related procedures, unfavorable host factors, or limited implant stability. Thus, infected sockets should not be categorically excluded, but they should be considered high-demand scenarios that require strict indication criteria and careful surgical execution.

In summary, immediate implant placement in infected extraction sockets may be clinically feasible in selected cases. However, its predictability depends heavily on anatomical, biological, surgical, and restorative conditions. The current evidence is encouraging but remains too heterogeneous to support a universal clinical protocol.

## Conclusions

This scoping review suggests that immediate implant placement in infected extraction sockets may be clinically feasible in carefully selected cases and may achieve favorable outcomes when case selection is strict, debridement is meticulous, and primary stability is achieved. However, this interpretation should be made with caution, because the available evidence remains heterogeneous in terms of infection definitions, socket characteristics, adjunctive protocols, loading strategies, follow-up periods, and reported outcomes. These limitations reduce direct comparability across studies and prevent the formulation of a standardized clinical protocol.

The main contribution of this review is to show that infected sockets should not be regarded as a uniform condition or as an absolute contraindication, but rather as complex clinical scenarios in which predictability depends on biological, anatomical, patient-related, and procedural factors. Better-designed comparative studies with standardized diagnostic definitions, protocol reporting, outcome measures, and longer follow-up are needed to clarify which clinical scenarios offer greater or lower predictability for immediate implant placement in infected extraction sockets. 

## Supplementary Material

Supplementary material 1

## Figures and Tables

**Table 1:** Table 1: General characteristics of the included studies.

**Study**	**Country**	**Design**	**Sample analyzed**	**Comparator**	**Follow-up**
Casco Silva & Silva Díaz 2025 [14]	Paraguay	Case report	1 patient/1 implant	None	3 years
Medikeri *et al*. 2018 [15]	India	Prospective study	8 patients/12 implants	None	12 months
Basualdo *et al*. 2018 [16]	Chile	Case report	1 patient/1 implant	None	21 months
Mattos *et al*. 2018 [17]	Brazil	Case report	1 patient/2 implants	None	24 months
Crippa *et al*. 2023 [18]	Italy/Spain	Retrospective cohort	149 implants	Infected laser-treated sites vs conventional implants in edentulous sites	≥1 year (up to >4 years)
Çolak & Demirsoy 2023 [9]	Turkey	Retrospective study	69 patients/124 implants	Three infected-site surgical subgroups	Not clearly stated; retrospective follow-up
Elaskary *et al*. 2024 [10]	Egypt	Prospective cohort	26 patients/41 implant sites reported	Infected vs non-infected type II sockets	12 months
Hita-Iglesias *et al*. 2016 [5]	Spain	Split-mouth controlled study	60 patients/168 implants	Chronic periapical pathology vs healthy sockets within same patients	1 year post-loading
Zuffetti *et al*. 2017 [6]	Italy	Multicenter retrospective study	369 patients/527 implants (193 infected, 334 non-infected)	Infected vs non-infected sites	Mean ~50 months
Crespi *et al*. 2017 [19]	Italy	Prospective study	60 patients/60 implants	Debridement vs no debridement of reactive tissue	12 months
Gomes *et al*. 2017 [20]	Brazil	Prospective study	14 patients (full-arch mandibular rehabilitation)	Longitudinal within-cohort microbiological/clinical follow-up	8 months of function
Crippa *et al*. 2020 [21]	Italy/Spain	Case report	1 patient/1 implant	None	5 years
Aljaky *et al*. 2024 [22]	Egypt	Prospective study	14 patients/14 implants	None	Up to 6 months after final loading (staged protocol)
Agustín-Panadero *et al*. 2015 [23]	Spain	Case report	1 patient/1 implant	None	12 months
da Silva *et al*. 2023 [24]	Brazil	Retrospective study	186 patients/423 implants (215 infected, 208 non-infected)	Chronic apical periodontitis vs non-infected sites	≥12 months after loading
Kakar *et al*. 2020 [8]	India	Retrospective cohort	68 patients/126 implants (110 analyzed)	Single infected-site cohort	Mean 48.8 months
Zohary *et al*. 2025 [25]	Iran	Randomized clinical trial	210 patients/210 implants	PRF vs CGF vs control	2 years
Anitua *et al*. 2016 [26]	Spain	Retrospective cohort	30 patients/43 implants	Single infected-site cohort	Mean 6 years
Narad *et al*. 2018 [27]	India	Prospective study	15 patients/24 implants	None	24 months
Wang *et al*. 2019 [28]	China	Case report	1 patient/1 implant	None	10 months
Muñoz-Cámara *et al*. 2020 [7]	Spain	Prospective cohort	100 patients/100 implants (50 infected, 50 controls)	Acute periapical pathology vs non-infected sockets	12 months
Sasmal *et al*. 2019 [29]	India	Case series	3 patients/3 implants	None	~1 year (ongoing follow-up)
Fang *et al*. 2021 [30]	China	Case report	1 patient/1 implant	None	12 months
Yang *et al*. 2023 [31]	China	Retrospective study	97 patients/97 implants (52 CAP, 45 no-CAP)	Mandibular molars with vs without CAP	5 years

Sample analyzed is reported as presented in the original studies and may refer to patients, implants, or implant sites. Follow-up is shown as reported by the source articles. CAP: Chronic apical periodontitis; no-CAP: No chronic apical periodontitis.

**Table 2:** Table 2: Clinical context, decontamination strategies, adjunctive measures, and restorative/loading protocols.

**Study**	**Infection/socket context**	**Anatomical site**	**Debridement/decontamination**	**Adjunctive measures**	**Loading/restoration**
Casco Silva & Silva Díaz 2025 [14]	Infected socket after vertical fracture	Maxillary lateral incisor/esthetic zone	Extraction and socket management as case protocol	Prosthetic rehabilitation	Delayed prosthetic rehabilitation
Medikeri *et al*. 2018 [15]	Periapical infection/chronic periapical lesions	Mixed anterior/posterior sites	Mechanical debridement + irrigation + antibiotic protocol	PRF + DFDBA	Loaded after 3 months
Basualdo *et al*. 2018 [16]	Infected site with active fistula and buccal plate loss	Anterior esthetic zone	Atraumatic extraction and local debridement	Ice-cream-cone GBR + collagen membrane + xenograft + L-PRF	Not immediately loaded
Mattos *et al*. 2018 [17]	Infected sockets with buccal bone resorption and apical periodontitis	Mandibular anterior esthetic area	Extraction, debridement and GBR-oriented socket preparation	Guided bone regeneration + fibrin biomaterial	Immediate provisionalization
Crippa *et al*. 2023 [18]	Post-extraction infected sockets	Mixed sites	Curettage + Er,Cr:YSGG laser decontamination	Protocol-based site management	Not primary focus
Çolak & Demirsoy 2023 [9]	Chronic periapical pathology	Mixed sites incl. GBR and sinus lift cases	Curettage/debridement and conventional infected-socket management	GBR and/or sinus lift in selected groups	Not primary focus
Elaskary *et al*. 2024 [10]	Type II infected compromised sockets with active signs of infection	Esthetic-zone compromised sockets	6-day protocol + VST	Cortical membrane + bone graft / VST	Immediate implant protocol within VST
Hita-Iglesias *et al*. 2016 [5]	Chronic periapical lesions	Upper incisors, canines and premolars	Careful selection, extraction and site preparation	Standard immediate implant protocol	Delayed loading
Zuffetti *et al*. 2017 [6]	Chronic periodontal or endodontic infection	Mixed sites across 5 centers	Proper preliminary site debridement + saline irrigation	Immediate/early loading allowed	Immediate or early loading in subset
Crespi *et al*. 2017 [19]	Asymptomatic apical periodontitis	Incisors, canines and premolars	Immediate placement with or without lesion tissue debridement	CBCT follow-up	Loaded after 3 months
Gomes *et al*. 2017 [20]	Chronic periodontal infection/ history of chronic periodontitis	Mandibular full-arch immediate implants	Extraction and immediate implant placement under periodontal control	Fixed complete-arch mandibular prostheses	Immediate/early full-arch loading
Crippa *et al*. 2020 [21]	Periapical periodontitis in esthetic zone	Maxillary lateral incisor/esthetic zone	Er,Cr:YSGG laser decontamination after extraction	Bio-Oss + resorbable membrane	Immediate placement (restoration not key focus)
Aljaky *et al*. 2024 [22]	Infected fresh extraction sockets/nonrestorable teeth	Mixed sites	Socket management plus topical ozonated olive oil	Ozonated olive oil	Two-stage; loaded after abutment healing
Agustín-Panadero *et al*. 2015 [23]	Periapical infection due to vertical fracture	Maxillary first premolar	Extraction and infected-socket preparation	Immediate provisional crown using extracted crown	Immediate loading
da Silva *et al*. 2023 [24]	Chronic apical periodontitis	Mixed sites	Thorough curettage and saline irrigation	Implant-level and patient-level risk analysis	Immediate or delayed loading depending case
Kakar *et al*. 2020 [8]	Previously infected sockets (endo/periodontal/mixed/trauma)	Maxilla and mandible	Er,Cr:YSGG laser decontamination + meticulous debridement + antibiotics	In situ hardening alloplastic graft	Non-submerged; loaded after mean 136 days
Zohary *et al*. 2025 [25]	Periapical lesions in previously infected sites	Mixed sites	Extraction and periapical lesion removal	PRF or CGF	Immediate implant placement; restorative timing per protocol
Anitua *et al*. 2016 [26]	Infected extraction sockets in anterior maxilla	Anterior maxilla	Socket curettage + PRGF; antibiotics	PRGF and immediate loading	Immediate loading
Narad *et al*. 2018 [27]	Subacute periodontal, perio-endo, chronic periapical infection, periodontal cyst, infected traumatic teeth	Mixed sites	Meticulous debridement under pre- and post-operative antibiotics	Standard immediate implant protocol	Not detailed as immediate loading
Wang *et al*. 2019 [28]	Periapical infection with residual bone height <1mm beneath sinus	Maxillary second molar with sinus involvement	Debridement and endoscope-guided transcrestal sinus management	PRF-only graft + endoscopic sinus floor elevation	Delayed crown placement
Muñoz-Cámara *et al*. 2020 [7]	Acute periapical pathology	Immediate implants with provisionalization	Meticulous curettage/debridement + chlorhexidine + saline irrigation	Immediate prosthetic provisionalization	Immediate provisionalization
Sasmal *et al*. 2019 [29]	Periapically infected sites	Anterior/premolar sites	Full-thickness flap, curettage and saline irrigation; antibiotics	GBR/xenograft/resorbable membrane in selected cases	Loaded after 3 months
Fang *et al*. 2021 [30]	Periapical infection with labial bone deficiency	Maxillary central incisor/esthetic zone	Extraction, local debridement and socket reconstruction	PRF + Bio-Oss + collagen membrane (modified GBR)	Definitive crown after 6 months
Yang *et al*. 2023 [31]	Chronic apical periodontitis in mandibular molars	Mandibular molars	Extraction and standard immediate implant management	Quantitative radiographic bone analysis	Restoration per protocol	

Infection/socket context, debridement/decontamination strategies, adjunctive measures

**Table 3:** Table 3: Main outcomes and reported complications in infected sockets.

**Study**	**Main outcomes in infected sockets**	**Complications/notes**
Casco Silva & Silva Díaz 2025 [14]	Successful osseointegration with favorable esthetic and functional outcome	No major complications reported
Medikeri *et al*. 2018 [15]	Implant survival 91.7%; improved gingival esthetics; non-significant crestal bone changes	One implant failure/one facial bone loss case
Basualdo *et al*. 2018 [16]	Resolution with buccal plate regeneration and stable peri-implant condition	No major complications reported
Mattos *et al*. 2018 [17]	Uneventful functional rehabilitation without relevant peri-implant bone loss	No painful symptoms, infection or mobility
Crippa *et al*. 2023 [18]	Only one failure in test group; no significant MBL difference versus controls	Low failure; protocol-dependent peri-implantitis prevention emphasized
Çolak & Demirsoy 2023 [9]	Overall success 95.6%; lower success when simultaneous sinus lift was required	Smoking and surgical complexity associated with lower success
Elaskary *et al*. 2024 [10]	100% survival; no significant bone or soft tissue differences versus non-infected group except mesial papilla	Minimal mucosal recession
Hita-Iglesias *et al*. 2016 [5]	Overall survival 98.2%, but significantly lower survival in pathological sites (90.8%) than controls (98.1%)	Some failures linked to excessive bone loss or lesion recurrence
Zuffetti *et al*. 2017 [6]	Cumulative survival 98.4% in infected vs 97.9% in non-infected sites; no significant difference	All failures occurred within first year
Crespi *et al*. 2017 [19]	100% survival; absence of apical radiolucency in both groups; no meaningful between-group differences	No increase in complications
Gomes *et al*. 2017 [20]	No major clinical complications; stable microbiological profile and compatible marginal bone behavior	No clinically relevant biological complications reported
Crippa *et al*. 2020 [21]	Successful long-term outcome without peri-implantitis or peri-implant bone loss	None reported
Aljaky *et al*. 2024 [22]	100% survival with favorable clinical healing and increased alveolar bone height on CBCT	No major complications reported
Agustín-Panadero *et al*. 2015 [23]	No biological or mechanical complications; high patient satisfaction	None reported
da Silva *et al*. 2023 [24]	Overall survival 91%; infection itself not significantly associated with failure	Short implants and heavy smoking predicted failure
Kakar *et al*. 2020 [8]	95.45% survival after loading	Five failures among analyzed implants
Zohary *et al*. 2025 [25]	PRF and especially CGF improved pain, BOP, esthetics and implant health versus control; successful osseointegration reported	Control group included compromised/satisfactory survival states
Anitua *et al*. 2016 [26]	No implant failures; success rate 93%; mean proximal bone loss 1.42 mm	Three veneer fractures
Narad *et al*. 2018 [27]	All implants stable at 24 months; crestal bone loss settled at first thread	No signs of mobility or ongoing infection
Wang *et al*. 2019 [28]	Stable hard and soft tissues; successful crown placement	None reported
Muñoz-Cámara *et al*. 2020 [7]	No significant clinical or radiographic differences versus controls; no retrograde peri-implantitis in infected group	No relevant biological complications reported
Sasmal *et al*. 2019 [29]	Cases showed favorable clinical and radiographic healing without evident bone loss at 1 year	None reported
Fang *et al*. 2021 [30]	Stable bone and soft tissue with satisfactory esthetic outcome	None reported
Yang *et al*. 2023 [31]	No significant differences in bone density, bone mass change, complications or survival between CAP and no-CAP groups	No excess complications attributable to CAP

Main outcomes and complications were summarized from the original studies and harmonized for co

**Figure 1:**
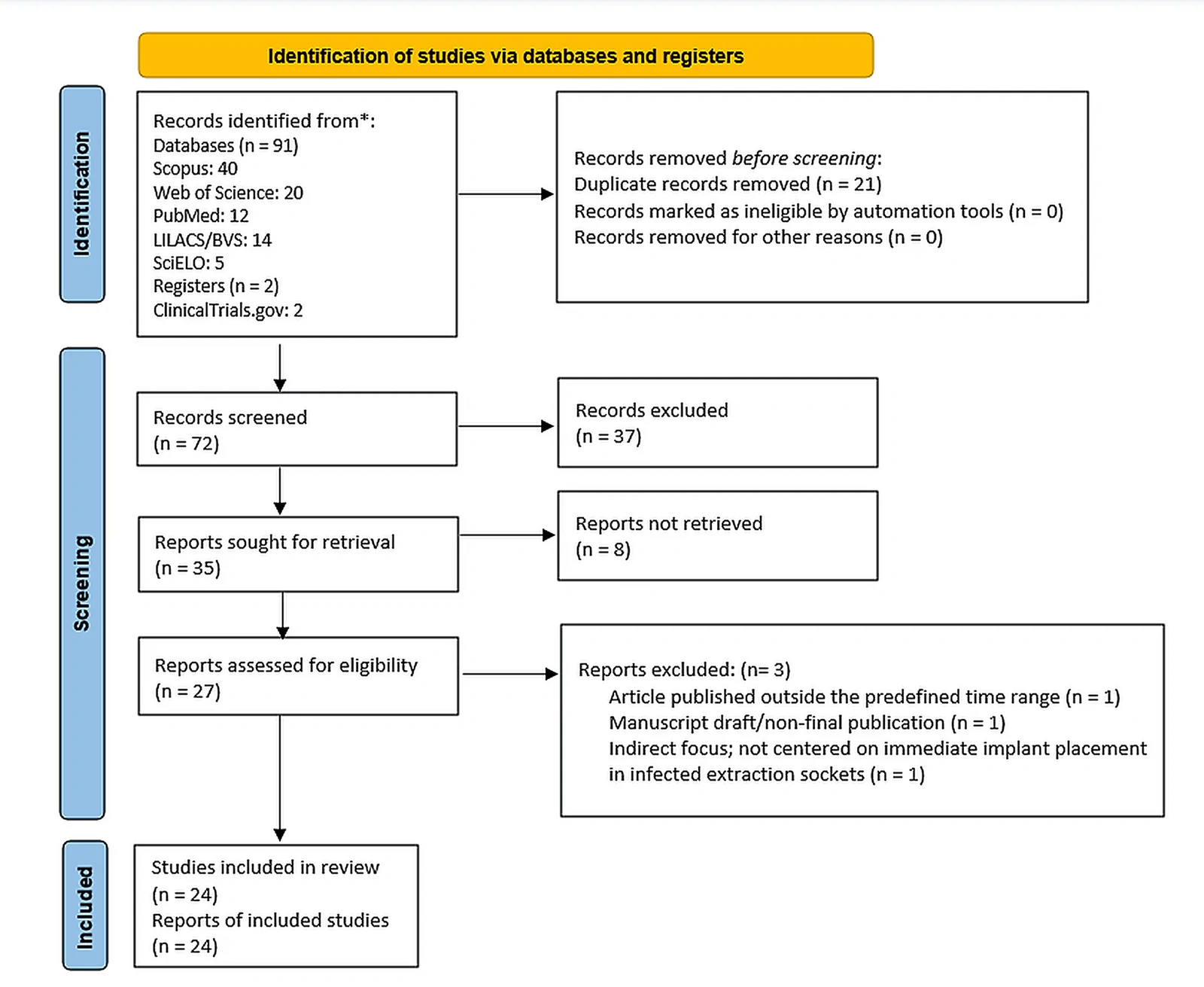
Fig. 1: Flow diagram of the study selection process, reported in accordance with PRISMA-ScR.

**Figure 2:**
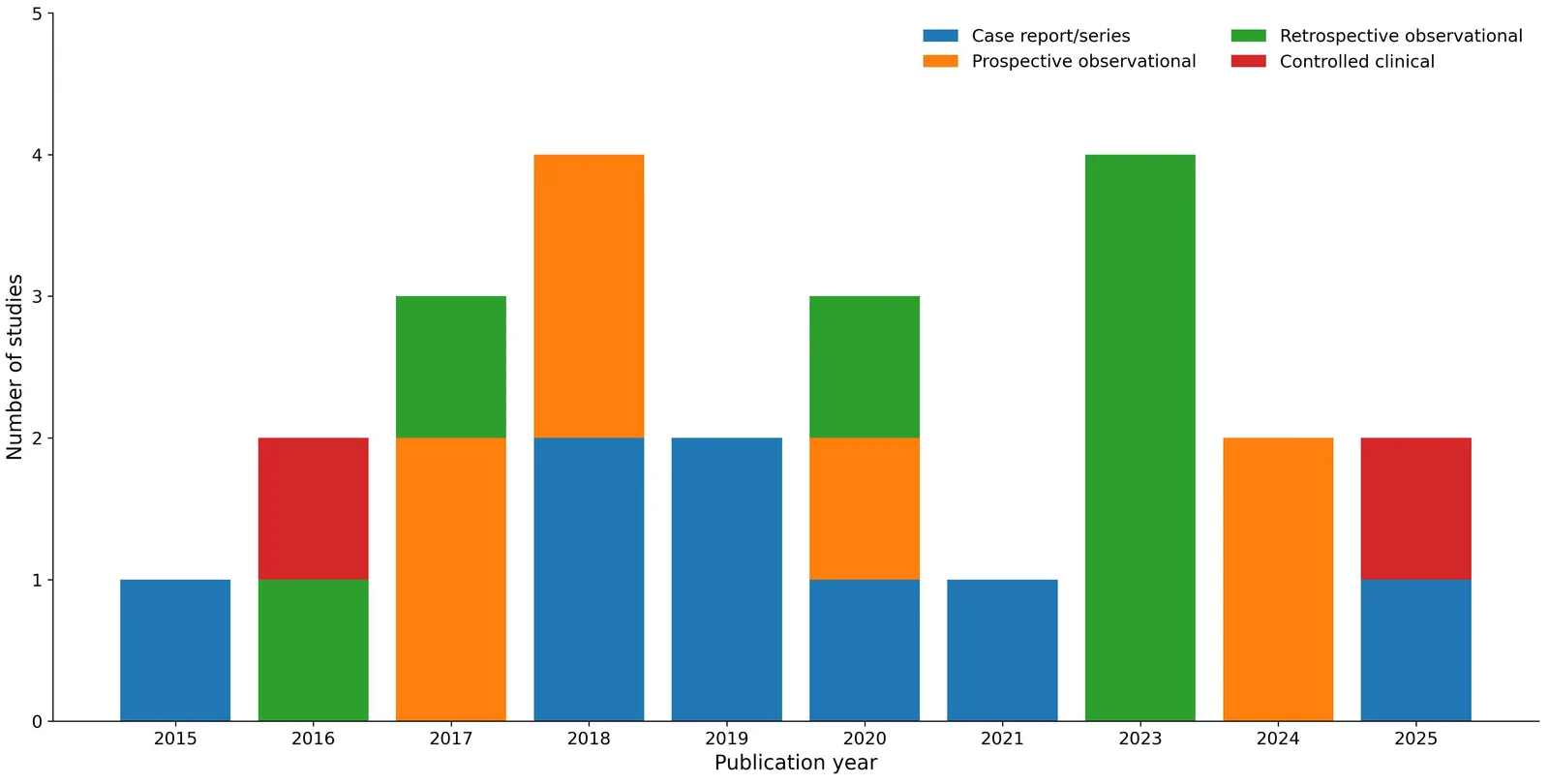
Fig. 2: Distribution of the included studies by publication year and study design.

**Figure 3:**
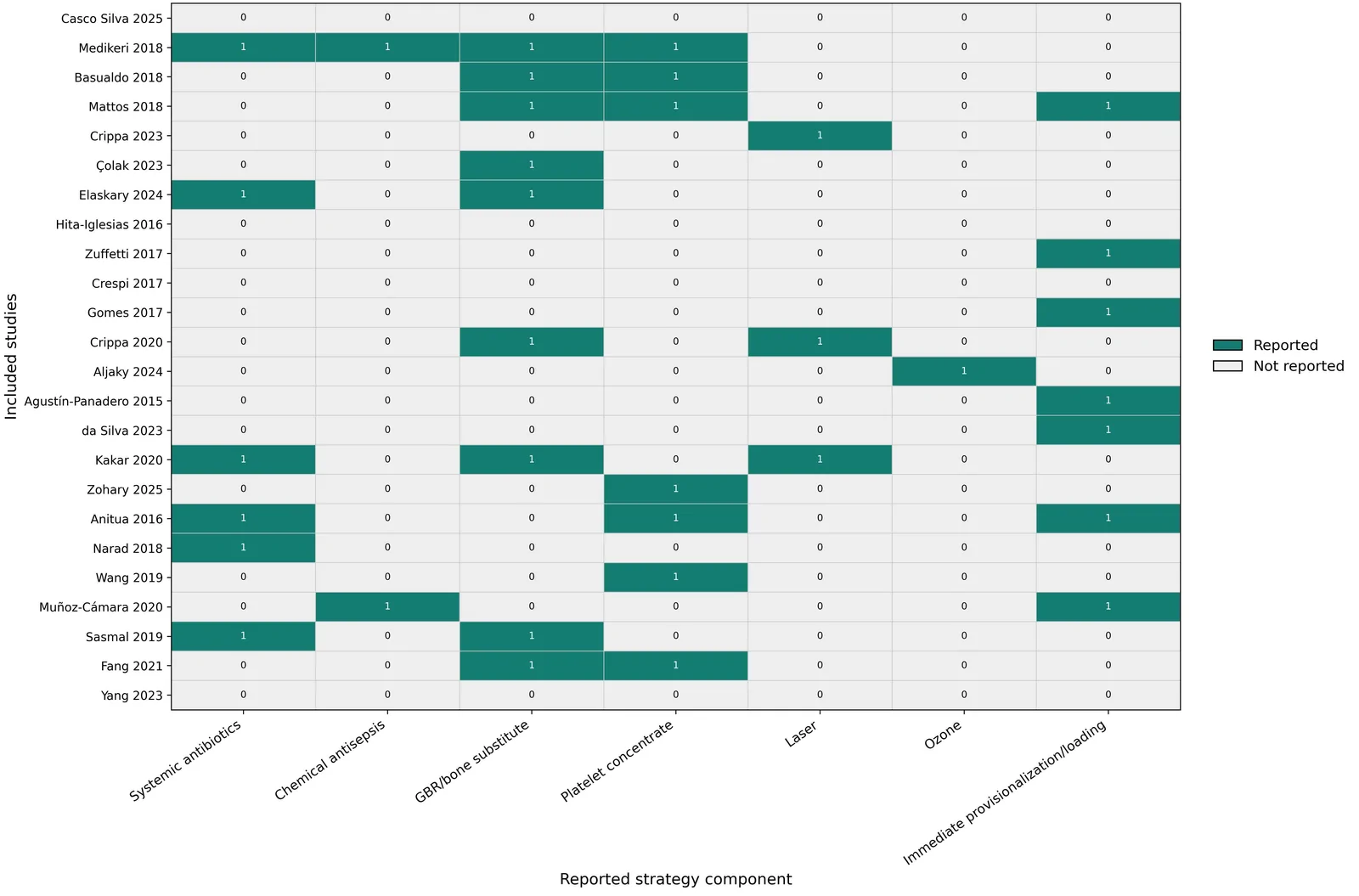
Fig. 3: Study-level heatmap of reported adjunctive management strategies across the included studies. Cells indicate whether each strategy component was explicitly reported and should not be interpreted as evidence of comparative effectiveness.

## Data Availability

None.
