# Supplementary material for: Immediate implant placement in infected extraction sockets: A scoping review of case selection, decontamination protocols, clinical outcomes and complications
Source: Med Oral Patol Oral Cir Bucal. 2026 Jun 28;31(5):e758–71. doi: 10.4317/medoral.28256 (PMC13636454; doi:10.4317/medoral.28256)
Supplement: Supplementary file 1 [file medoral-31-05-e758-s001.pdf]

Supplementary Appendix S1. Full-text eligibility decisions

Topic: Immediate implant placement in infected extraction sockets. **Full-text reports assessed:** 27; included: 24; excluded: 3. **Eligibility criteria applied:** human clinical studies published between 2015 and 2026, immediate post-extraction implant placement, clearly described infected socket or periapical pathology, and original clinical data.

Table S1. Included full-text studies

Study	Design	Reason for inclusion
Casco Silva & Silva Díaz, 2025 [14]	Case report	Immediate implant in an infected socket with 3-year follow-up; direct clinical evidence for the review question.
Medikeri et al., 2018 [15]	Prospective study	Immediate implants in sockets with periapical infection; PRF + DFDBA protocol and clinical/radiographic outcomes.
Basualdo et al., 2018 [16]	Case report	Immediate implant in an infected site using the ice-cream-cone technique and L-PRF; relevant regenerative protocol.
Mattos et al., 2018 [17]	Case report	Immediate rehabilitation in infected alveolar sockets with 24-month follow-up and no relevant complications.
Crippa et al., 2023 [18]	Retrospective cohort	Immediate implants in infected sites decontaminated with Er,Cr:YSGG laser compared with controls; survival and radiographic data.
Çolak & Demïrsoy, 2023 [9]	Retrospective study	Immediate implants in sockets with periapical pathology; includes clinically relevant surgical subgroups.
Elaskary et al., 2024 [10]	Prospective cohort	Infected vs non-infected type II sockets treated with vestibular socket therapy; hard- and soft-tissue outcomes.
Hita-Iglesias et al., 2016 [5]	Split-mouth study	Direct comparison of sites with chronic periapical pathology and healthy sockets within the same patients.
Zuffetti et al., 2017 [6]	Multicenter retrospective study	Large cohort comparing infected and non-infected post-extraction sites; implant survival outcomes.
Crespi et al., 2017 [19]	Prospective study	Immediate implants in sockets with asymptomatic apical periodontitis; fully aligned with the review focus.
Gomes et al., 2017 [20]	Original clinical study	Immediate implants in patients with chronic periodontal infection; clinical and microbiological outcomes.
Crippa et al., 2020 [21]	Case report	Infected esthetic-zone site treated with laser decontamination and long-term follow-up; maps adjunctive management.
Aljaky et al., 2024 [22]	Prospective study	Immediate implants in infected sockets using ozonated olive oil; clinical and radiographic outcomes.
Agustín-Panadero et al., 2015 [23]	Case report	Immediate implant and immediate loading in a socket with periapical infection; meets the eligibility criteria.
da Silva et al., 2023 [24]	Retrospective study	Analyzes failures of immediate implants in infected and non-infected sites and identifies relevant predictors.
Kakar et al., 2020 [8]	Consecutive cohort	Immediate implants in infected sockets using a standardized decontamination and grafting protocol.
Zohary et al., 2025 [25]	Clinical trial	PRF, CGF, and control groups in previously infected sites; 2-year follow-up and detailed clinical outcomes.
Anitua et al., 2016 [26]	Retrospective cohort	Immediate implants in infected sockets with immediate loading and long-term follow-up.
Narad et al., 2018 [27]	Original clinical study	Immediate implants in prepared/debrided infected sockets with at least 24-month clinical and radiographic follow-up.
Wang et al., 2019 [28]	Case report	Immediate implant in a periapical lesion site associated with transcrestal sinus elevation and PRF.
Muñoz-Cámara et al., 2020 [7]	Prospective cohort	Acute periapical pathology vs non-infected sites, both managed with immediate provisionalization.
Sasmal et al., 2019 [29]	Case series	Immediate implant rehabilitation in periapically infected sites; useful for mapping clinical approaches.
Fang et al., 2021 [30]	Case report	Immediate implant in an esthetic-zone periapical infection with PRF; reports regenerative and esthetic outcomes.
Yang et al., 2023 [31]	Retrospective study	Mandibular molars with vs without chronic apical periodontitis; 5-year comparative outcomes.

Table S2. Excluded full-text reports

No.	Study/report	Reason for exclusion
1	Blus et al., 2013	Outside the predefined publication window (2015-2026).
2	Cho et al. (manuscript draft)	Manuscript draft/non-final document; not identifiable as a formal final publication.
3	Wang et al., 2017	Main focus was complete prosthetic rehabilitation; did not centrally evaluate immediate implant placement in infected extraction sockets.

Note. This supplementary table summarizes the full-text eligibility decisions for the 27 reports assessed. Terminology was harmonized for clarity and compact presentation.
